# Setting the global research agenda for community health systems: literature and consultative review

**DOI:** 10.1186/s12960-019-0362-8

**Published:** 2019-03-21

**Authors:** Smisha Agarwal, Karen Kirk, Pooja Sripad, Ben Bellows, Timothy Abuya, Charlotte Warren

**Affiliations:** 1 Population Council, 4301 Connecticut Avenue NW, Suite 280, Washington DC, 20009 United States of America; 2 Department of International Health, Johns Hopkins Bloomberg School of Public Health, 615 N Wolfe Street, Baltimore, MD 21205 United States of America; 3 Population Council, Avenue 5, 3rd Floor, Rose Avenue, Nairobi, Kenya

**Keywords:** Community health systems, Community health workers, Frontline health workers, Primary health care, Literature review

## Abstract

**Background:**

Globally, there is renewed interest in and momentum for strengthening community health systems, as also emphasized by the recent Astana Declaration. Recent reviews have identified factors critical to successful community health worker (CHW) programs but pointed to significant evidence gaps. This review aims to propose a global research agenda to strengthen CHW programs.

**Methods and results:**

We conducted a search for extant systematic reviews on any intermediate factors affecting the effectiveness of CHW programs in February 2018. A total of 30 articles published after year 2000 were included. Data on research gaps were abstracted and summarized under headings based on predominant themes identified in the literature. Following this data gathering phase, two technical advisory groups comprised of experts in the field of community health—including policymakers, implementors, researchers, advocates and donors—were convened to discuss, validate, and prioritize the research gaps identified.

Research gap areas that were identified in the literature and validated through expert consultation include selection and training of CHWs, community embeddedness, institutionalization of CHW programs (referrals, supervision, and supply chain), CHW needs including incentives and remuneration, governance and sustainability of CHW programs, performance and quality of care, and cost-effectiveness of CHW programs. Priority research questions included queries on effective policy, financing, governance, supervision and monitoring systems for CHWs and community health systems, implementation questions around the role of digital technologies, CHW preferences, and drivers of CHW motivation and retention over time.

**Conclusions:**

As international interest and investment in CHW programs and community health systems continue to grow, it becomes critical not only to analyze the evidence that exists, but also to clearly define research questions and collect additional evidence to ensure that CHW programs are effective, efficient, equity promoting, and evidence based. Generally, the literature places a strong emphasis on the need for higher quality, more robust research.

**Electronic supplementary material:**

The online version of this article (10.1186/s12960-019-0362-8) contains supplementary material, which is available to authorized users.

## Background

There is renewed global interest and momentum to strengthen community health systems. In a 2008 World Health Organization (WHO) report, “Primary Health Care, Now More than Ever,” released on the 30th anniversary of the Alma Ata Conference, the critical role of community health workers (CHWs) was emphasized. The report revisited the major challenges, policies, and recommendations to accelerate delivery of primary health care (PHC), including through the strategic deployment of CHWs [1]. With advent of the Sustainable Development Goals (SDGs) in 2015, the need to integrate community health approaches in local health systems became paramount. The revitalization of interest in community health systems has been accompanied by international conviction, increased investments, and a preponderance of new systematic reviews to collate research findings and best practices [2–6].

In March 2017, the United States Agency for International Development (USAID) and the United Nations Children’s Fund (UNICEF), in collaboration with the World Health Organization (WHO), the Bill & Melinda Gates Foundation, and USAID’s flagship Maternal and Child Survival Program (MCSP), hosted the Institutionalizing Community Health Conference in Johannesburg, South Africa [7]. The conference gathered delegations from 45 countries, including government officials, policymakers, and civil society leaders to focus on evidence that would inform national policies and optimize engagement with communities as resources for community health systems. Following the conference, UNICEF initiated a Community Health-Community of Practice (CH CoP) to maintain momentum on community health by bringing together implementers, policymakers, practitioners, and others to share challenges and insights to scale community health systems [8].

In addition to the investments and global meetings, over the last 5 years, a number of systematic reviews on various aspects of CHW programs have been undertaken. These include a WHO-commissioned series of systematic reviews for developing guidelines on community health worker programs and a special supplement of the *Journal of Global Health* that synthesized over 700 reports and studies on various aspects of community-based primary health care [9, 10]. Although most of these reviews have identified factors that are critical to successful CHW programs, they regularly point to a lack of rigor in study design, note the limitations in meta-analyzing impacts due to non-comparable measurements, and identify significant evidence gaps needing further research. Scott and colleagues recently published a review of systematic reviews of CHW programs that identified significant gaps in knowledge about CHW programs, and recommended that evidence needs to be contextualized and prioritized [2]. The WHO CHW guideline provides critical recommendations on key questions to strengthen community health worker programming and which have a more direct and policy-relevant agenda to optimize the design and performance of CHW programs.

As the global community reaffirms its commitment to primary health care in the Astana Declaration [11] and renews its commitment to support community health systems, it is crucial that there is a clear forward-looking agenda for research to support effective and scaled community health programs. The purpose of this review is to develop a global policy and practice-oriented research agenda for community health systems. We shift attention from whether CHW programs are effective to identifying which factors make CHW programs more effective. To prioritize areas for future research, our consultative efforts take a broader lens by undertaking a multi-pronged approach involving a review of available literature and a series of expert consultations including with ministries of health, which are often not reflected in more traditional reviews.

## Methods

Knowledge collation for this review was conducted in three stages. First, we conducted a search for extant systematic reviews and overview of reviews on factors affecting CHW program implementation and effectiveness published after year 2000. In community health, the historic perspective holds critical relevance given the limited progress and dearth of knowledge generation in the 1990s and the more recent revitalization of, and support for, community health over the last decade. In fact, much of the recent literature reiterates several gaps identified in the space nearly two decades ago. We used a “snowball” technique, where one article led to the discovery of additional reports. Reviews were included if they identified critical knowledge gaps in CHW program implementation. Reviews that limited their focus to the impact of the CHW programs on health outcomes were excluded. A total of 30 articles were included. Summary themes were identified inductively based on a detailed review of the papers, and data were abstracted under these core themes.

Second, to prioritize the identified research gaps within an operational context, in May 2018, representatives from seven implementing partners of the Integrating Community Health (ICH) Program (supported by USAID) and counterparts from their respective ministry of health (MOH) were convened at a 4-day workshop in Johannesburg, South Africa. Implementing ICH organizations include Save the Children, Bangladesh; Zanmi Lasante, Haiti; Aga Khan Foundation, Mali; Last Mile Health, Liberia; Humana People to People, Democratic Republic of Congo (DRC); Liverpool School of Tropical Medicine, Kenya; and Pathfinder International, Uganda [12]. MOH representatives were present from all countries except Bangladesh. Workshop participants represented countries at different stages of CHW program institutionalization, needs, and challenges. The preliminary list of research gaps from the literature review was presented, and attendees were asked to identify three priority research areas that would aid in scaling the community health system in their respective country.

Third, a technical advisory group (TAG) of 18 community health experts, including donors, implementers, researchers, and advocates, was convened in Washington, DC. In a priority-setting exercise, the TAG experts were asked to reflect on a list of 32 research gaps identified through the desk review and Johannesburg consultation. All respondents graded the importance of each research question (responses: Yes, Maybe, No) and identified five of the 32 research questions viewed as the highest priority for addressing knowledge gaps to aid in scaling community health systems. An additional file aligns the survey responses with the research areas identified from the literature (see Additional file 1).

The resulting priorities have been summarized below under predominant themes in the literature and include findings from all the research activities: the review of the literature as well as the two consultative meetings.

## Results

### Selection and training of CHWs

There is a lack of evidence on optimal processes for CHW selection and the impacts of different selection policies [13, 14]. For example, selecting CHWs with high educational requirements may prevent representation of certain communities [15], reduce community support for a CHW, and result in higher CHW attrition [16]. Traditionally, in small programs, CHW selection is informal, guided by local support and social norms. As programs grow, selection processes become more formal, with regional administrative selection requirements, which may undermine the community’s role. Leaving the selection process entirely to a local community can, however, be affected by local politics and traditions and lead to selection through sub-optimal criteria. Further research could test appropriate selection strategies under different stages of maturation.

There is also limited research on the optimal design of CHW programs’ accreditation and certification [2]. CHW training typically involves both a theoretical and practical component in the pre-service phase, as well as ongoing in-service training for skills updates and renewals. As a step towards CHWs’ institutionalization, several countries—notably Uganda, Haiti, Mali, and Liberia—are in the process of formalizing their training programs, with the intention of accrediting CHWs [17–21]. A recognized gap in the literature is the need for testing innovative approaches to CHW pre-service training and measuring how CHW baseline characteristic variations, such as gender and education, as well as the length of pre-service training, may affect CHW performance and patient outcomes [4, 22–26]. This all resonated with several policymakers as they expressed a need to understand models of training that will result in high CHW competency [21]. Additionally, there is a need to study the effectiveness of job aids during training [27, 28], including digital tools, compared to more traditional training methodologies [29]. National programs typically require rapid scale up of training infrastructure, using models such as training of trainers (ToT) [2]. More insights are needed around cost of training models as costs vary considerably by the mode of training, as well as the location (e.g., disbursed training close to the community versus bringing CHWs to centralized training facilities), and have significant bearing on how ministries can reasonably proceed [21]. Mediating performance and cost while balancing intensity and frequency of contact are critical considerations in CHW training design and policy [2].

Training content is another consideration, with tension between an emphasis on broad training to address social determinants of health with necessary community mobilization and counseling skills versus focused, competency-based biomedical training [13]. In addition to the type of work to be trained for, there also needs to be guidance on the volume of work that would contribute to CHW efficiency [6]. Some evidence suggests that CHW technical competency declines after training, and therefore follow on, regularly supervised practice, and mentored opportunities are needed [23, 30].

### Community embeddedness

A CHW’s level of *embeddedness* in a community is essential to her or his success. “Community embeddedness” can refer to the level of community CHW acceptance and investment, as well as the level of CHW engagement, including trust and empathetic relationships a CHW has with the community. From a research perspective, community embeddedness is difficult to study, as communities are heterogeneous with complex power dynamics [13]. The utility of community embeddedness comes from the assumption that a CHW with high levels of trust and empathetic relationships within the community is better positioned to enter community members’ homes and counsel them on sensitive health matters. Scott et al. state that acceptance from a community may affect CHW retention, motivation, performance, and accountability, but that there is minimal evidence on how to strengthen a CHW’s connection to the community [2]. What role, if any, does involving local communities in CHW selection and training, and do community structures play, in advancing community embeddedness and subsequent CHW performance and retention? [23, 26, 28, 30–33]. Inversely, how does the work of the CHW empower communities to respond to traditional barriers to care-seeking, harmful practices and stigma? [15, 26, 27, 31, 34]. How do these relationships change, given differing contexts and settings? [29].

### Institutionalization of CHW programs

Like CHWs’ horizontal integration with the community, their vertical integration within the health system is integral to their success. Research is needed to identify and test existing mechanisms to integrate and institutionalize CHWs within the formal health system [23, 32] and the extent to which institutionalization enhances performance [28–30]. Although research has not conclusively defined all components of health systems integration, several points emerged as important, including referrals, supervision, and supply chain.

### Referrals

There is a need to test whether models of “shared care” involving referral and counter-referral between communities and health facilities can influence CHW performance, especially where communications and transportation systems are weak [16, 23, 27]. Bosch-Capblanch and colleagues suggest the need for more rigorous research comparing models that link peripheral health services with a central managerial unit [35].

### Supervision

Numerous programs and research studies emphasize adequate supervision of CHWs for effective performance. Evidence gaps about effective supervisory approaches (e.g., type and frequency) continue, however [24–27, 30, 36]. The need to study supervisory mechanisms also emerged as important needs of the ministries of health in Bangladesh, Haiti, and DRC during the ICH meeting [21]. Evidence suggests supervision quality may be more important than frequency, up to a point [29, 37, 38]. Perceived supervision, integrating the CHW perspective of lived experienced of supervisory quality, is also integral to explore across country contexts [11]. Given this, the comparative efficacy of different models of supervision, such as peer, group, and community supervision, and self-assessment, through checklists and a combination of these approaches, needs to be tested within the broader health system using robust study designs [28, 37]. Regular supervision contributes substantially to costs associated with CHW programs, and the cost-effectiveness of various supervisory approaches should also be evaluated [29]. In addition to supervision, alternate accountability mechanisms should be explored further, from a health systems perspective [33].

### Supply chain

Strategies to improve the routine availability of medical commodities emerge in the literature as another research priority [16] and were also emphasized by participants at the ICH meeting [21]. In the recent years, the use of digital tools to report on stock levels with CHWs and the use of data dashboards to improve transparency of district-level stocks have gained support. Further support is needed for studies to understand how these tools can be best integrated with existing inventory management systems and used by CHWs [39].

### CHW needs including incentives and remuneration

Historically, scaled CHW programs have suffered from significant attrition over time and low productivity [2], with several reasons cited including inadequate attention to CHW concerns about their pre-service training, supervisory support, financial and non-financial incentives, satisfaction with their role, and professional development opportunities [25, 40]. As with other cadres of workers, CHWs’ incentive satisfaction is closely linked to their motivation, but research on this matter continues to be piecemeal, small in scale, and contextual, with limited generalizability [25, 41, 42]. Existing research suggests that formal salaries for a large cadre of CHWs may be financially unsustainable at a national scale in most low- and some middle-income countries; however, a combination of financial and non-financial incentives such as t-shirts/caps, other social recognition, certifications, resource availability, and positive working relationships may improve CHW motivation and reduce attrition [23, 40, 41, 43]. The ethical dimensions of asking CHWs to volunteer their time need to be considered, and more research is needed on an appropriate combination of these incentives (appropriate training/certification, career opportunities, social recognition, performance-based financing, allowance and salaries) which could be applicable across contexts and commensurate with their job demands [6, 23, 26–28, 34, 40, 42, 44]. Further research is also needed on payment systems that could reward accountability [32]. This information needs to be contextualized to specific country settings to understand what levels, methods, and types of incentives are cost-effective, given a country’s gross domestic product (GDP) and health expenditure trends [16, 43]. These results need to be disaggregated by gender, and more research is needed on meeting the needs of, in some cases, a predominantly female CHW workforce [21, 28]. Incentives’ effects on CHWs’ motivation, as well as their effects on performance and patient outcomes, have scarce empirical evidence [23, 31].

Attention also needs to be paid to the risk of exploiting CHWs, particularly women [2]. The emphasis on achieving Sustainable Development Goals (SDGs) and universal health care (UHC) has resulted in expanded CHW roles beyond health care, across a variety of sectors including education and agriculture; however, limited progress has been made in institutionalizing compensatory mechanisms for their time.

### Governance and sustainability of CHW programs

Much remains unknown about how CHW programs, once scaled nationally, can be sustained [21]. Questions remain about what policy and governance structures should ensure their sustainability [21]. Short-term donor funding and different stakeholder agendas can disrupt programs’ sustainability [23, 45]. Research is needed to understand the context and conditions in which it makes sense to implement and integrate CHW programs [16]. Large-scale implementation of programs should be accompanied by research to determine the contextual factors that affect CHW programs’ performance and scale [6, 26, 28, 30, 32, 36].

The effect of decentralization of governance on CHW program implementation has emerged as a critical area for further research [2], especially in contexts like Kenya where the recent devolution has resulted in the simultaneous implementation of different CHW models across its 47 counties [21]. In Bangladesh, with a pluralistic health system, several cadres of CHWs exist; each focused on specific health areas, with some overlapping activities. While the reasons for multiple models of CHW programs are different in Kenya and Bangladesh, the research needs to focus on identifying which of these models are most effective and what types of governance structures can support them [21]. For example, in Bangladesh, it is important to understand the level of coordination required across ministries to harmonize job descriptions and activities of different cadres of health workers [21]. Structural changes in these programs’ governance may also influence CHW motivation and performance [31]. In additional to national and regional level governance, there is also a need to study the effect of civil society players in advocating for CHW programs and improving CHW accountability [21].

### Performance and quality of care

How do each of these factors—training, community embeddedness, institutionalization, governance—affect CHWs’ productivity and quality of care, especially as programs scale up? [22, 25, 38]. Traditionally, clients’ satisfaction with CHW services has been used as a proxy measure for CHW quality of care; however, as programs scale, measuring their services’ technical quality becomes critical [15]. An equity perspective in the study of quality of care is integral. Does quality of care provided vary by type of community group, wealth, or gender [6, 15, 16, 21, 32], and how can the services provided by CHWs be channeled to reduce stigma and adapt socio-cultural norms? [36]. What types of drugs can CHWs safely administer, and what might be the implications for antibiotic resistance or a patient’s informed choice of contraceptives? [16]. With the advent of new technologies in the community health space, it becomes important to test innovative technology-based approaches (such as the use of pre-filled injection devices and mobile job-aids) on quality of care [32]. Review studies have pointed out the challenges in assessing the impact of CHW programs on quality services, in part due to low-quality evidence [23, 30] and lack of standardized metrics in quality assessment [28]. To the furthest extent possible, future research should employ validated quality metrics, and where possible use longitudinal research designs, to assess the effects on quality of services over time [27]. CHW performance also needs to be assessed in specific populations such as adolescents [26], refugees and displaced populations [46], and in epidemic settings such the Ebola epidemic in DRC and Liberia [21].

### Cost-effectiveness of CHW programs

There is some evidence of cost-effectiveness of CHW programs within the context of delivering services for tuberculosis, HIV, pediatric asthma, and management of malaria [6, 25–27, 47]. However, given the challenges of assessing cost-effectiveness of integrated multi-component interventions with varying typologies, much of the data continues to be of low quality, insufficient, and limited in its generalizability [6, 25–27, 47]. The need for costing data and cost-effectiveness evidence is a common refrain from policymakers, and it emerged as one of the most important questions by government representatives participating in the ICH partners meeting. Some priority areas included the need to assess the cost of the different models and conduct comparative cost-effective work in Bangladesh and Kenya and assess the cost of innovative models such as CHWs using technology to improve quality of care in Uganda and Liberia [21].

Further studies on costs and cost-effectiveness are needed for specific interventions delivered by CHWs such as vaccination programs, malaria prevention and treatment, and mental health programs [24, 32, 34, 48], as well as comparing CHW models that are single-disease focused versus “generalist” CHWs that offer a range of community-based services [26]. Cost and cost-effectiveness studies may be guided by certain principles to be more operationally relevant across contexts—for example, varying the timeframe to explore the impact of the program over short and long terms [23], incorporating detailed costing of personnel (e.g., including supervisory costs) and other resources associated with the intervention, and using multiple comparative scenarios as alternatives [28, 29, 34]. While challenging to implement, costing studies should also consider costs against the quality of service [29]. Vaughan et al. suggest that given the level of embeddedness of CHW programs within the community, cost-effectiveness studies also need to capture societal costs and benefits, including aspects such as social capital, trust, and costs to clients [47].

### Prioritization of research areas based on consultations with technical advisory group (TAG)

Discussion with the TAG helped to prioritize and contextualize the research priorities identified through the literature review and consultations with ICH partners. There was consensus on the need to consider the community health system holistically—beyond CHW programs—in that the ability of CHWs to perform effectively often depends on their community and health systems environments. Additional priorities not highlighted in the literature emerged—research on the effectiveness of CHW programs to address non-communicable diseases and the use of digital tools to strengthen CHW programs.

The priority setting survey yielded a range of responses from the 18 experts convened and confirmed many of the priorities that were also identified through the literature.

Of the 32 research areas, the following questions received the highest consensus.

- What are effective and efficient supervisory and monitoring structures (e.g., peer, group, community, health facility supervisory models) to improve CHW performance within a specified health area?
- How and to what extent are digital technologies helpful as a component of supervision and monitoring of CHWs?
- What policies, financing, and governance structures are required to support and ensure sustainability of CHW programs?
- Which CHW models are cost-effective and improve quality of care?
- What combination of training, incentives, and career growth opportunities increase CHW motivation and retention?

## Discussion

There is a wealth of knowledge within the community health system space that dates to the 1970s. However, despite over four decades of research, critical gaps persist. While research has typically focused on the evaluation of the effectiveness of small-scale CHW programs, there is a need to view programs from a broader lens to understand what intermediate factors are associated with successful implementation of these programs at scale. Additionally, given the rapid evolution of technology, as well as the epidemiological transition to chronic disease in many low- and middle-income countries, research needs to focus on new areas and new models of service delivery by CHWs. There is also a need for higher quality and more robust research that answers operational questions, as ministries tackle the challenges of scaling these programs. In addition to new research content and enhanced quality, future studies should account for the complexity of integrated CHW programs—considering that these programs operate within the governance and health ecosystem that supports them.

Several reasons are likely for the persistent gaps in evidence about CHW programs. First, in most countries CHW programs tend to be fragmented, often with varied or inconsistent implementation by state and non-governmental actors. Historically, the selection criteria, training, and support systems for CHWs have not been standardized. This has downstream effects on the ability to generate effectiveness evidence that can be generalized across programs. With the push towards institutionalizing and professionalizing CHW programs, standardized protocols are also being put in place, which should support a long-term cohesive vision for these programs. Second, given the complexity of these programs—defined by the level of community and health system embeddedness, comprehensiveness of health services delivered, and expected effect on community mobilization and empowerment—they are methodologically difficult to assess using singular research designs. In a recent analysis, George and colleagues [49] recommend that a broader evidence agenda, comprised of a balance of research designs to capture the community context, including the level of community embeddedness, is critical to advance community programs [2]. Third, the scope and existence of CHW programs evolves with the needs of the communities and with the focus of international donor support. While responsiveness to community needs is critical, donors and the global community should be cautious in advocating for an expanded scope of CHW activities.

Given the renewed international interest and investment in CHW programs, as highlighted by the Astana Declaration, we use a multi-pronged approach to not only define a broad future research agenda, but also to prioritize research areas where resources should be directed. In early 2017, the Kampala statement highlighted the potential of CHWs in achieving several SDGs targeting outcomes of poverty, hunger and food security, health, gender equality, and clean water and sanitation [50]. While this holistic perspective on community health is important, it also risks spreading the research agenda too thin. A focused, responsive research agenda is critical as policymakers grapple with the challenges of scaling up these programs with limited resources. Research needs to be embedded within policy and program development from the onset and answer operational questions around effective and cost-effective strategies that policymakers can use to adapt programs.

This review leverages the wealth of information on CHW program effectiveness to identify and summarize ongoing research gaps, as well as validates the known gaps in literature, and identifies new priorities through consultations with a range of stakeholders, including country policymakers, implementers, and other global experts. The country priorities presented in this review are derived from workshop-based discussions with implementers and policymakers; a more in-depth country level analysis is needed to identify summative research priorities for any specific country.

Strengthening CHW programs is particularly pertinently to the advancement of the three principles of equity, quality and protection against financial risk to achieve universal health coverage at the grassroots [51]. The current review of persisting gaps aligns with many of the prior reviews of CHW programs and identifies areas where robust research is needed to recognizably move the global agenda forward to institutionalize successful strategies for sustainable CHW programs.

## Conclusion

The current review of persisting gaps aligns with many of the prior reviews of CHW programs and identifies areas where robust research is needed to recognizably move the global agenda forward to institutionalize successful strategies for sustainable CHW programs. Given the renewed international interest and investment in CHW programs and community health systems continue to grow, we use a multi-pronged approach not only to define a broad future research agenda, but also to prioritize research areas where resources should be directed.

## Additional file

Additional file 1: Priority research gaps identified by the literature: mapping TAG survey responses. Description of data: Table linking research gaps emerging from literature to the responses identified in the survey responses of members of the Technical Advisory Group. (DOCX 29 kb)

## Abbreviations

CH CoP: Community Health-Community of Practice
CHW: Community health worker
DRC: Democratic Republic of Congo
GDP: Gross domestic product
ICH: Integrating Community Health
MCSP: Maternal and Child Survival Program
MOH: Ministry of health
PHC: Primary health care
SDGs: Sustainable Development Goals
TAG: Technical advisory group
ToT: Training of trainers
UNICEF: United Nations Children’s Fund
USAID: United States Agency for International Development
WHO: World Health Organization
