# Supplementary material for: Setting the global research agenda for community health systems: literature and consultative review
Source: Hum Resour Health. 2019 Mar 21;17:22. doi: 10.1186/s12960-019-0362-8 (PMC6429801; doi:10.1186/s12960-019-0362-8)
Supplement: Supplementary file 1 — Priority research gaps identified by the literature: mapping TAG survey responses. Description of data: Table linking research gaps emerging from literature to the responses identified in the survey responses of members of the Technical Advisory Group. (DOCX 29 kb) [file 12960_2019_362_MOESM1_ESM.docx]

*Priority research gaps identified by the literature: mapping TAG survey responses*

| ***Research Question*** | ***Number of articles*** | ***Article citations*** | ***% agreement on TAG survey**** |  |
| --- | --- | --- | --- | --- |
| **Selection and training of CHWs** | | | | |
| 1.   What policy criteria, institutional, and community engagement processes determine optimal and equitable CHW selection? Are selection criteria restrictive and do they lead to inequities? | 7 | Campbell et al., 2011; Bhutta et al., 2009 ; Haines et al., 2007 ; South et al., 2011 ; Gilmore & McAuliffe, 2013 ; Koon et al., 2013 ; Fulton et al., 2011 | 22% |  |
| 2.   How do training approaches with varied modality, duration, frequency, and theory-practice mix comparatively influence CHW competence? | 4 | Scott et al., 2018 ; Campbell et al., 2011; Bosch-Capblanch & Marceau, 2014 ; Koon et al., 2013 ; | 56% |  |
| 3.   What training methodologies and tools (such as job aids) are most cost-effective for national scale up (e.g. localized, train-the-trainer, centralized)? | 4 | Scott et al., 2018 ; Bosch-Capblanch & Marceau, 2014 ; Kaman-Yanni et al., 2012 ; Rowe et al., 2005 | 50% |  |
| 4.   What training models can be adapted to different contexts (rural, urban, high/low resourced country, humanitarian) and how? | 3 | Fulton et al., 2011 Rowe et al., 2005 | 33% |  |
| **CHW needs and rights including financial and non-financial incentives** | | | | |
| 5. What level and type of incentives are cost-effective for countries at different stages of development and health expenditure? | 10 | Scott et al., 2018 ; Campbell et al., 2011; Bhutta et al., 2009 ; Bosch-Capblanch & Marceau, 2014 ; Dawson et al., 2013 ; Franco et al., 2002 ; Glenton et al., 2013 ; Koon et al., 2013 ; Fulton et al., 2011 ; Dieleman et al., 2008 | 50% |  |
| 6.  What combination of training, incentives, and social protections (e.g. safety, workload) affect CHW performance? | 7 | Scott et al., 2018 ; Campbell et al., 2011; Sibley et al., 2012 ; Haines et al., 2007 ; Franco et al., 2002 ; Glenton et al., 2013 ; Fulton et al., 2011 | 62% |  |
| 7.  How do gender, age, and other vulnerabilities of CHWs affect their needs and improve their job performance? | 4 | Scott et al., 2018 ; Bosch-Capblanch & Marceau, 2014 ; Glenton et al., 2013 ; Koon et al., 2013 | 28% |  |
| **Community embeddedness** | | | | |
| 8.     How do we measure community embeddedness and trust in CHWs in the context of community health systems? | 2 | Scott et al., 2018 ; Campbell et al., 2011 | 22% |  |
| 9.       How does community embeddedness of a CHW affect his or her motivation, performance, retention and accountability? | 5 | Scott et al., 2018 ; Lewin et al., 2010 ; Bosch-Capblanch & Marceau, 2014 ; Franco et al., 2002 ; Glenton et al., 2013 | 44% |  |
| 10.   How and to what extent does CHW engagement in the community lead to increased community trust in the health system and improved care-seeking behaviors? | 4 | Scott et al., 2018 ; McCollum, 2016 ; Glenton et al., 2013 ; Koon et al., 2013 ; | 44% |  |
| 11.   How do CHW-community relationships vary in different geographic and socio-political settings? | 5 | Campbell et al., 2011 ; McCollum, 2016 ; Lewin et al., 2010 ; Franco et al., 2002 ; Rowe et al., 2005 | 11% |  |
| **Institutionalizing CHWs** | | | | |
| 12.   What policies and mechanisms exist for integrating CHWs into health systems? | 6 | Scott et al., 2018 ; Lewin et al., 2010 ; Franco et al., 2002 ; Glenton et al., 2013 ; Koon et al., 2013 ; Rowe et al., 2005 | 56% |  |
| 13.   How does the type of CHW integration enhance program implementation and CHW performance? | 3 | Scott et al., 2018 ; Haines et al., 2007 ; Glenton et al., 2013 | 39% |  |
| 14.   How do models of “shared care” involving CHWs and a mix of facility-based providers influence CHW performance, community-facility linkage, and referral? | 5 | Scott et al., 2018 ; Molyneux et al., 2012 ; Dawson et al., 2013 ; Franco et al., 2002 ; Rowe et al., 2005 | 56% |  |
| 15.   What are appropriate mechanisms for referrals from the community to the facility, especially in contexts where communications and transportation systems are weak? | 4 | Sibley et al., 2012 ; Haines et al., 2007 ; Glenton et al., 2013 ; Corluka et al., 2009 | 50% |  |
| 16.   How can routine supplies be made regularly available and equitably distributed to CHWs? | 2 | Jaskiewicz and Tulenko, 2012; Haines et al., 2007 ; | 56% |  |
| 17.   *What are effective and efficient supervisory and monitoring structures (e.g. peer, group, community, health facility supervisory models) to improve CHW performance within a specified health systems context?* | 6 | Scott et al., 2018 ; Hill et al., 2014 ; Jaskiewicz and Tulenko, 2012; Bosch-Capblanch & Marceau, 2014 ; Bosch-Capblanch Garner, 2008; Rowe et al., 2005 | 78% |  |
| *18.   How and to what extent are digital technologies helpful as a component of supervision and monitoring of CHWs?*** | 0 |  | 83% |  |
| **Governance and sustainability of CHW programs** | | | | |
| *19.   What policies, financing, and governance structures are required to support and ensure sustainability of CHW programs?* | 6 | Scott et al., 2018 ; Lewin et al., 2010 ; Molyneux et al., 2012 ; Franco et al., 2002 ; Glenton et al., 2013 ; Rowe et al., 2005 | 78% |  |
| 20.   Under what conditions does it make sense to implement CHW programs, and at what point (if any) should they be phased out? | 1 | Zulu et al., 2014 | 11% |  |
| 21.   The nature and extent to which short-term donor funding, different stakeholder perspectives (including a dynamic civil society), and coordination of CHW models affect sustainability of community health systems? | 4 | Scott et al., 2018 ; Bosch-Capblanch & Marceau, 2014 ; Glenton et al., 2013 ; Zulu et al., 2014 | 28% |  |
| 22.   How does context – national/sub-national changes, humanitarian/emergency situations, geographical settings – influence CHW programming and ensure accountable community health systems? | 7 | Scott et al., 2018 ; Molyneux et al., 2012 ; Franco et al., 2002 ; Glenton et al., 2013 ; Rowe et al., 2005 ; Corluka et al., 2009 ; Zulu et al., 2014 | 33% |  |
| **Performance and quality of care** | | | | |
| *23.   What combination of training, incentives, and career growth opportunities increase CHW motivation and retention?* | 4 | Scott et al., 2018 ; Jaskiewicz and Tulenko, 2012; Glenton et al., 2013 ; Kaman-Yanni et al., 2012 | 72% |  |
| 24.   How is the technical quality of care measured for CHW programs as they are scaled up? | 3 | Scott et al., 2018 ; McCollum, 2016 ; Bosch-Capblanch Garner, 2008 | 50% |  |
| 25.   Does technology have a role to play in scaling CHW programs and improving performance? | 3 | Lewin et al., 2010 ; Rowe et al., 2005 ; Fulton et al., 2011 | 50% |  |
| 26.   Does the quality of care provided by CHWs vary by population group (socioeconomically distinct, adolescents, refugees, displaced populations)? | 1 | McCollum, 2016 | 17% |  |
| 27.   What types of CHW activities reduce disease-related stigma and empower communities to adapt socio-cultural norms? | 1 | Kok et al., 2015b | 28% |  |
| 28.   How does the performance and quality of services provided by CHWs vary over time, since initial implementation? | 2 | Bhutta et al., 2009 ; Glenton et al., 2013 | 50% |  |
| **Cost-effectiveness of CHW programs** | | | | |
| *29.   Which CHW model is cost-effective and improves quality of care?* | 7 | Scott et al., 2018 ; Bhutta et al., 2009 ; Haines et al., 2007 ; Lewin et al., 2010 ; Rowe et al., 2005 ; Fulton et al., 2011 ; Corluka et al., 2009 | 78% |  |
| 30.   How do costs and cost-effectiveness vary over time between focused and generalist CHW models? | 6 | Scott et al., 2018 ; Lewin et al., 2010 ; Glenton et al., 2013 ; Kaman-Yanni et al., 2012 ; Koon et al., 2013 ; Corluka et al., 2009 | 56% |  |
| 31.   What are cost-effective supervisory approaches to support CHW programs? | 2 | Jaskiewicz and Tulenko, 2012; Bosch-Capblanch Garner, 2008 | 67% |  |
| 32.   What are the societal costs and benefits of CHW programs and how are these associated with community embeddedness? | 1 | Vaughan et al., 2015 | 39% |  |

*18 Respondents total; this agreement refers to those who all responded “yes” as to this question being a priority question.

**This was raised from ICH partners and in the TAG discussions
